# Multiple benefits of alloparental care in a fluctuating environment

**DOI:** 10.1098/rsos.172406

**Published:** 2018-02-21

**Authors:** Sarah Guindre-Parker, Dustin R. Rubenstein

**Affiliations:** 1 Department of Ecology, Evolution and Environmental Biology, Columbia University, New York, NY, USA; 2 Department of Ornithology, National Museums of Kenya, Nairobi, Kenya; 3 Center for Integrative Animal Behavior, Columbia University, New York, NY, USA

**Keywords:** alloparental care, helping behaviour, cooperative breeding, environmental unpredictability, reproductive success, load-lightening

## Abstract

Although cooperatively breeding vertebrates occur disproportionately in unpredictable environments, the underlying mechanism shaping this biogeographic pattern remains unclear. Cooperative breeding may buffer against harsh conditions (hard life hypothesis), or additionally allow for sustained breeding under benign conditions (temporal variability hypothesis). To distinguish between the hard life and temporal variability hypotheses, we investigated whether the number of alloparents at a nest increased reproductive success or load-lightening in superb starlings (*Lamprotornis superbus*), and whether these two types of benefits varied in harsh and benign years. We found that mothers experienced both types of benefits consistent with the temporal variability hypothesis, as larger contingents of alloparents increased the number of young fledged while simultaneously allowing mothers to reduce their provisioning rates under both harsh and benign rainfall conditions. By contrast, fathers experienced load-lightening only under benign rainfall conditions, suggesting that cooperative breeding may serve to take advantage of unpredictable benign breeding seasons when they do occur. Cooperative breeding in unpredictable environments may thus promote flexibility in offspring care behaviour, which could mitigate variability in the cost of raising young. Our results highlight the importance of considering how offspring care decisions vary among breeding roles and across fluctuating environmental conditions.

## 1. Background

Despite decades of research on the ecological factors that shape animal sociality, the precise role that ecology plays in shaping the occurrence or evolution of cooperative breeding behaviour remains equivocal [1]. Broad-scale comparative analyses have demonstrated that cooperatively breeding species of birds [2,3] and mammals [4] occur more frequently in unpredictable environments where annual rainfall is low and highly variable through time. This pattern has long been taken as evidence that unpredictable environments shape the evolution of cooperative breeding [2,3], though recent work suggests that cooperation may also facilitate the colonization of variable and harsh environments [5]. But what is the underlying mechanism to explain these biogeographic patterns? The ‘hard life hypothesis' suggests that fluctuating environments represent an ecological constraint [6], where harsh conditions limit independent breeding opportunities or successful offspring rearing. Thus, cooperative breeding may serve to buffer against these harsh conditions, allowing for successful reproduction when it would otherwise be impossible to raise young without the assistance of alloparents. The ‘temporal variability hypothesis’ goes a step further and argues that in addition to buffering against harsh conditions, alloparents may also serve to provide substantial benefits under benign conditions [2]. Thus, in fluctuating environments where the quality of any given year is unpredictable, breeding cooperatively may allow for both successful reproduction when conditions are poor and for sustained breeding or improved reproductive success when favourable conditions occur. Therefore, having alloparents to help rear young in fluctuating environments may provide benefits exclusively under harsh conditions (hard life hypothesis), or under both harsh and benign conditions (temporal variability hypothesis). Although these hypotheses are similar in predicting that alloparents provide benefits in harsh environments, only the temporal variability hypothesis predicts that alloparents also provide benefits under benign conditions. Understanding the benefits of cooperative breeding in unpredictable environments represents a critical area of research to explain the role of ecology in shaping the distribution of cooperatively breeding species globally [1].

Previous efforts examining the benefits of cooperative breeding behaviour have focused primarily upon the reproductive benefits of having a greater number of alloparents. Typically, the presence of alloparents can increase the number or quality of young produced annually by enhancing the quality of offspring care provided [7,8], or the number of breeding attempts undertaken by a breeding pair [9]. However, alloparents may also allow breeders to lower their current investment in offspring care, which can increase their own body condition [10] or survival [11–13]. The care decision rules that breeders follow in response to receiving alloparental assistance will in large part shape the type of benefits that arise. When alloparents provide offspring care, breeders may choose to respond by either (1) *decreasing* the amount of care they provide themselves, thereby reducing the cost of reproduction that they incur (termed compensatory care, or ‘load-lightening’ [14,15]), or (2) *maintaining* their contribution to offspring care, thereby increasing the cumulative care that breeders and alloparents provide to the young (termed ‘additive care’ [15]). These two parental care strategies represent the care decision rules that breeders can choose to use in response to alloparental care and may, therefore, mask the current reproductive benefits of having alloparents. For example, alloparents may not increase reproductive success at a nest if breeders opt to lighten their load in favour of potential future fitness benefits such as increased survival or future reproduction. It is, therefore, important not only to consider current reproductive and load-lightening benefits, but also the mechanism through which these benefits arise (i.e. additive care versus load-lightening) to fully understand the benefits of receiving alloparental care in unpredictable environments.

Empirical tests of the hard life and temporal variability hypotheses have generated mixed results. For species living in fluctuating environments, alloparents have been shown to increase reproductive success only under harsh environmental conditions (consistent with the hard life hypothesis) [7,16–18], only under benign conditions [19], or across harsh and benign environmental conditions (consistent with the temporal variability hypothesis) [20,21]. Part of this inconsistency in how alloparental care influences fitness in fluctuating environments may exist because environmental conditions could contribute to shaping care decision rules, and thus the type of benefits that breeders gain from having alloparents. Yet to our knowledge, no study has investigated whether individuals adjust their offspring care decision rules (i.e. additive care versus load-lightening) according to fluctuating environmental conditions, despite the fact that doing so is critical to understanding how environmental quality shapes the benefits of breeding cooperatively. For example, breeders would be expected to favour load-lightening when the relative costs of offspring care are elevated [22], which should occur when environmental conditions are harsh [23]. As a result, alloparents may be less likely to raise breeders' reproductive success during these harsh periods and may instead only raise breeders’ reproductive success under benign conditions. In this way, fluctuating environmental conditions may drive a trade-off between the current and future fitness benefits of having alloparents at the nest, and this trade-off may be mediated by environmentally driven changes in offspring care decision rules. Thus, although environmental conditions appear to influence the incidence and biogeographic distribution of cooperative breeding behaviour in birds, the underlying benefits—either reproductive or load-lightening—of breeding cooperatively in fluctuating environments remain poorly understood.

Here we examine how temporal variability in rainfall, as well as spatial variability in territory quality, shape the benefits of having alloparents in the cooperatively breeding superb starling, *Lamprotornis superbus*. Superb starlings inhabit one of the world's most unpredictable environments—the African savannah—where rainfall varies in intensity and duration within and among years [24]. Significant variation also exists in grass cover across territories, which together with rainfall shapes the availability of insects used to provision young [24,25]. To identify the benefits of alloparental care in environments that fluctuate both in time and in space, we test whether alloparents increase the number of nestlings fledged and/or provide load-lightening primarily (i) under harsh environmental conditions (as predicted by the hard life hypothesis), (ii) under both harsh and benign environmental conditions (as predicted by the temporal variability hypothesis) or (iii) under benign conditions (as has been found in acorn woodpeckers, *Melanerpes formicivorus* [19]). Alternatively, it is possible that alloparents provide no benefit, or that the benefits they provide are not related to fledging success or load-lightening. This research will improve our understanding of the mechanism through which fluctuating environmental conditions can alter the benefits of breeding cooperatively, thereby helping to explain why cooperatively breeding species may occur more frequently in unpredictable environments.

## 2. Material and methods

### 2.1. Study system

Superb starlings are plural cooperative breeders that live in large social groups of up to 50 individuals, including multiple breeding pairs (typically three or four [24]). A population of uniquely marked superb starlings in seven social groups was monitored continuously from 2002 to 2015 at the Mpala Research Centre, Kenya (0°17′ N, 37°52′ E). In this population, superb starlings breed twice per year, during the short (October–November) and long rains (March–June) [26]. The number of alloparents at a nest is variable (range 1–14; [24]) because some group members choose to forgo both breeding and alloparental care in a given breeding season (termed non-breeder/non-helpers). As a result, the number of alloparents at a particular nest can vary considerably and independently of group size (see electronic supplementary material, S1 and table S1). Alloparents range in age from juveniles to adults, can be of either sex, and can vary in their relatedness to breeding individuals [27].

### 2.2. Measuring environmental variation

We measured variability in precipitation by quantifying pre-breeding and breeding rainfall for each breeding season using an automated Hydrological Services TB3 Tipping Bucket Rain Gauge [26]. We calculated breeding rainfall as the sum of daily rainfall from March to June for the long rains and from October to November for the short rains. We also calculated pre-breeding rainfall, which occurs during the dry seasons immediately preceding each rainy season, from July to September for the short rains and from December to February for the long rains. Both pre-breeding and breeding rainfall play important yet distinct roles in shaping superb starling behaviour and reproductive success. That is, pre-breeding rainfall is thought to influence the proportion of first-time breeders within social groups [28], whereas breeding rainfall drives the availability of insects used to provision young [24].

We also examined how variability in territory quality could affect reproductive or load-lightening benefits. Territory quality was defined as the amount of grass cover on a territory, calculated as the long-term average of the proportion of dropped pins that touched vegetation from monthly vegetation transects performed between 2008 and 2015 [25]. Territory grass cover increases with rainfall similarly for all territories, and both rainfall and grass cover are positively correlated to the availability of insects used to provision young [24]. However, grass cover provides additional information on local insect availability that is not captured by rainfall alone because territories have repeatable spatial differences in grass cover that remain consistent across months, seasons and years [26].

Although we included continuous measures of rainfall and territory quality in our analyses, we considered harsh conditions as seasons with below average pre-breeding (less than 110 mm) or breeding rainfall (less than 230 mm), and low-quality territories as ones with below average grass cover (less than 64%). In contrast, we considered benign conditions as those with above average rainfall (pre-breeding ≥ 110 mm; breeding rainfall ≥ 230 mm) and high-quality territories as those with above average grass cover (≥ 64%). Thus, although we focused upon temporal environmental variation (rainfall), we also considered spatial environmental variation (grass cover) because they are linked in this system.

### 2.3. Monitoring reproduction

We monitored 716 nesting attempts over the course of this study by visiting nests every 1 to 3 days to determine clutch size and the fate of each nestling until fledging [29,30]. The two primary causes of fledging failure in superb starlings are nestling starvation and predation [24]. Starvation was identified when chicks were found dead in or near the nest, or when one previously underweight nestling disappeared. Conversely, predation usually left visible damage to the nest and often resulted in the entire clutch disappearing at once. Although nest predators are quite diverse (e.g. birds, baboons, snakes, squirrels, genets and mice [31]) and vary in the type of damage they cause, the most commons signs of nest predation included tearing of the nest, blood in the nest or nestling bands found near the nest. We assumed that nestlings were depredated when previously heavy nestlings went missing suddenly and earlier than the typical fledging age. Predation events were further differentiated from fledging via observations in the current breeding season, as juvenile superb starlings remain with their parents in their natal groups up to 2 years post-fledging [24]. In the rare cases where we could not determine what happened to one or more nestlings, they were classified as missing and excluded from our analyses (less than 2.5% of cases from more than 1000 hatchlings).

### 2.4. Measuring parental care behaviour

Active nests were observed for 2 h approximately twice per week, which is comparable to other behavioural studies of cooperative breeding birds [32–34]. Of the 716 nests monitored during this study, we performed behavioural observations at 162 nests (due to nest predation or abandonment at the others). Focal observations were collected for a minimum of 2 h per nest (the average nest was observed for nearly 6 h: mean ± s.e. = 5.6 ± 0.15 h). The identity of each bird arriving within 20 m of the nest was determined with a spotting scope, and the time of arrival and departure from the nest area was recorded. We also recorded entry and exit times from the nest, and whether birds delivered food into the nest. When food was brought to the nest, we recorded the size of food items delivered to young (relative to bill length). However, we found that mothers, fathers and alloparents delivered similar sized food items to nestlings (mean ± s.e.: fathers 1.2 ± 0.09; mothers 1.1 ± 0.06, alloparents 1.2 ± 0.09), making provisioning rate a stronger indicator of offspring care than prey item size (as in [35]). We used these observations to calculate two behaviours that reflect investment in offspring care: nest guarding and offspring provisioning [31]. Guarding was defined as the proportion of time an individual spent within 20 m of the nest (but not inside) relative to the length of the observation period. Time spent perched near the nest constitutes guarding in superb starlings because (1) perched individuals frequently make alarm calls when predators approach [24], and (2) alloparents rapidly attack predator models placed near the nest, performing approximately half of the attacks on the models [31]. Offspring provisioning rate was defined as the number of trips per hour where an individual delivered food into the nest.

### 2.5. Defining breeding roles

At each nest, we measured the guarding or provisioning rate of the mother, father and the average alloparent (averaged from all alloparents at the nest). We chose to average alloparent behaviour regardless of their relatedness to breeders, age or sex because previous work in this species found that these factors account for few differences in alloparental care behaviour [31]. Breeders at each nest were identified by a combination of behavioural and genetic analyses. Briefly, the mother was identified as the bird incubating at a nest, while the social father was identified as the male closely following the incubating female [27]. We also confirmed parentage using microsatellite markers [36] from nestlings and adults using DNA extracted from blood samples [25,27]. Conversely, all additional individuals provisioning offspring and/or spending time guarding the nest were classified as alloparents. We then counted the number of alloparents observed at each nest to assess whether individuals of each breeding role adjusted their behaviour based upon the size of the alloparent contingent. A preliminary investigation revealed that we were more likely to detect a greater number of alloparents at nests observed for a longer cumulative period of time (see electronic supplementary material, S2 and figure S2.1). To account for this, we calculated the residuals of the number of alloparents on observation time and used this as an index of the number of alloparents present at a nest (see electronic supplementary material, S2 and table S2). Larger positive residuals indicate that a nest had a larger contingent of alloparents than expected given the length of time we spent observing that nest, while more negative values indicated a smaller contingent of alloparents than expected based on observation length.

### 2.6. Statistical analyses

*Reproductive benefits of alloparental care*: We built four generalized linear mixed models (GLMMs) to examine how (i) clutch size, (ii) the number of fledglings, (iii) the number of hatchlings starved and (iv) the number of hatchlings depredated related to the number of alloparents across environmental conditions. Each model included pre-breeding rainfall, breeding rainfall, grass cover and the residual number of alloparents as fixed effects in addition to the interaction between the number of alloparents and each of the three environmental variables. We also included random effects of year and breeder ID in each model to control for repeated measures. We assumed a Poisson error distribution (log link function) to model clutch size, and a negative binomial error distribution (logit link function) with zero-inflation to model the number of fledglings, starved nestlings and depredated nestlings because these variables were highly skewed towards zero (due to the high occurrence of nesting failure) and were overdispersed.

*Load-lightening benefits of alloparental care*: We built four GLMMs for each of the two offspring care behaviours (guarding and provisioning) examining how environmental conditions or the relative number of alloparents at a nest altered the behaviour of (i) the group cumulatively, (ii) mothers, (iii) fathers or (iv) alloparents. The number of minutes spent nest guarding and the number of provisioning trips an individual performed were modelled using a negative binomial error distribution. GLMMs for mothers did not require zero-inflation, whereas GLMMs for fathers, alloparents or cumulative behaviour did. GLMMs for both behaviours and for all roles also included an offset accounting for the cumulative length of focal observations performed at each nest [37]. Each model included pre-breeding rainfall, breeding rainfall, grass cover, and the number of alloparents present as fixed effects, the interaction between the number of alloparents and the three environmental variables as fixed effects, as well as year and breeder ID as independent random effects.

All GLMMs were run in the package ‘glmmADMB’ v. 0.8.3.3 in R (v. 3.2.4 [38]). We originally included two additional fixed effects in all models—breeding season and social unit—but removed these variables because they were redundant with rainfall and grass cover, respectively. We then tested for multicollinearity among our remaining fixed effects and found that our predictor variables were not linearly related to one another (all variance inflation factors (VIF) < 2). Each continuous fixed effect was standardized before analyses (i.e. converted to *z*-score) to improve model convergence [39]. We checked the normality and homogeneity of GLMM residuals, which were suitable.

## 3. Results

### 3.1. Reproductive benefits

Clutch size did not vary with pre-breeding or breeding rainfall, grass cover, the relative number of alloparents at a nest, or any interaction between these variables (see electronic supplementary material, S3 and table S3.1). Instead, mothers consistently laid approximately three eggs (mean ± s.e. = 3.12 ± 0.03) regardless of environmental or social context. Similarly, environmental conditions were unrelated to the number of nestlings that fledged successfully. However, having a higher number of alloparents providing offspring care at a nest was associated with an increased number of young fledged (table 1). We found that the reproductive benefit of having alloparents at a nest did not vary across environmental conditions (i.e. interactions between pre-breeding rainfall, breeding rainfall or grass cover and the number of alloparents were not significant in our model) (see electronic supplementary material, S3 and table S3.1).

**Table 1.** Parameter estimates for a GLMM examining how environmental conditions, the relative number of alloparents at a nest, or the interaction between these variables shape the number of nestlings that fledged (*N* = 162). Asterisks highlight significant variables (*p* < 0.05). *p*-values and 95% Wald confidence intervals are presented in electronic supplementary material, table S3.3.

fixed effects	estimate ± s.e.	*Z*
intercept	0.41 ± 0.12	3.30*
pre-breed. rain	−0.13 ± 0.12	−1.07
breeding rain	0.13 ± 0.10	1.21
grass cover	0.03 ± 0.11	0.30
no. alloparents	0.82 ± 0.22	3.71*
pre-breed. rain × no. alloparents	0.11 ± 0.21	0.53
breeding rain × no. alloparents	0.32 ± 0.20	1.60
grass cover × no. alloparents	−0.20 ± 0.23	−0.90

random effects	variance ± s.d.	*N*
mother ID	0.0000007 ± 0.0008	65
father ID	0.0000001 ± 0.0003	59
year	0.000009 ± 0.003	13

**Figure 1.**
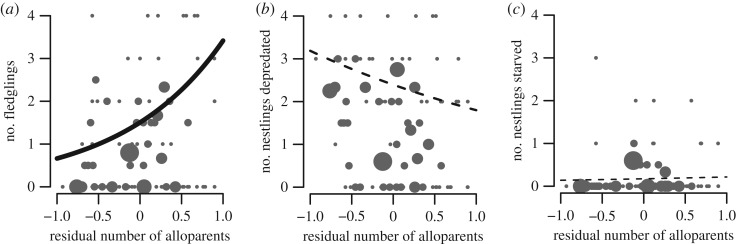
The number of alloparents present at a nest (*a*) increased the number of offspring fledged, (*b*) tended to decrease the number of nestlings that were depredated, but (*c*) was unrelated to the number of nestlings starved. Lines represent GLMM model predictions (solid, statistically significant; dotted, non-significant); data points represent mean values for each mother in a given year, where symbol size is proportional to the sample size.

Next, we examined potential causes underlying differences in fledging success. The number of nestlings that starved or were depredated at a nest was unrelated to pre-breeding rainfall, breeding rainfall or grass cover. The number of starved nestlings was also unrelated to the number of alloparents at a nest, though this analysis included nests that were later depredated (in contrast, previous work excluding nests that were depredated showed that alloparents do reduce nestling starvation in superb starlings [24]). The number of nestlings starved was unrelated to interactions between the number of alloparents and environmental conditions (see electronic supplementary material, S3 and table S3.1). However, the number of nestlings depredated decreased with a larger contingent of alloparents (though this effect was not statistically significant). This result was consistent across harsh and benign seasons as well as low- and high-quality territories, as interactions between environmental variables and the number of alloparents were not significant (see electronic supplementary material, S3 and table S3.1). Thus, alloparents primarily provide reproductive benefits to breeding superb starlings by reducing nestling depredation events (figure 1).

**Figure 2.**
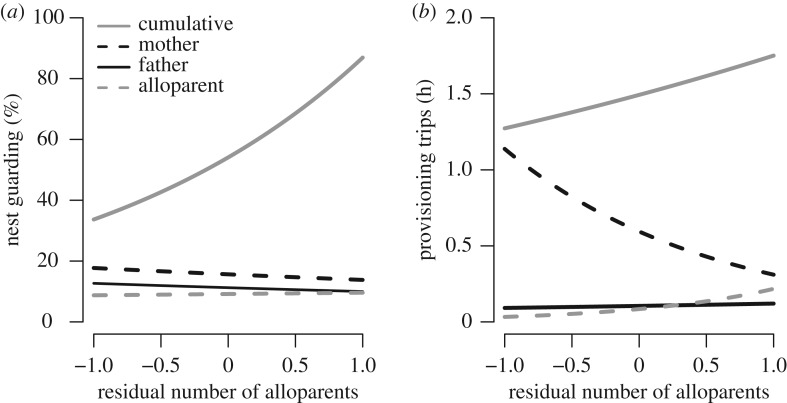
(*a*) Cumulative nest guarding (solid grey line) increased with the number of alloparents present at a nest. Mothers (dashed black line) and fathers (solid black line) did not alter their nest guarding behaviour as the number of alloparents increased, but individual alloparents (dashed grey line) were more likely to perform greater nest guarding when there were more alloparents present. Similarly, (*b*) the cumulative provisioning rate by all individuals increased with the number of alloparents present. Mothers experienced load-lightening because they provisioned at a lower rate when there were more alloparents at a nest. Conversely, fathers and alloparents increased their provisioning rates when the number of alloparents at a nest increased. Lines represent GLMM model predictions, where other parameters are set to their mean values. Data for each breeding role are pictured in greater detail in figure 3.

**Figure 3.**
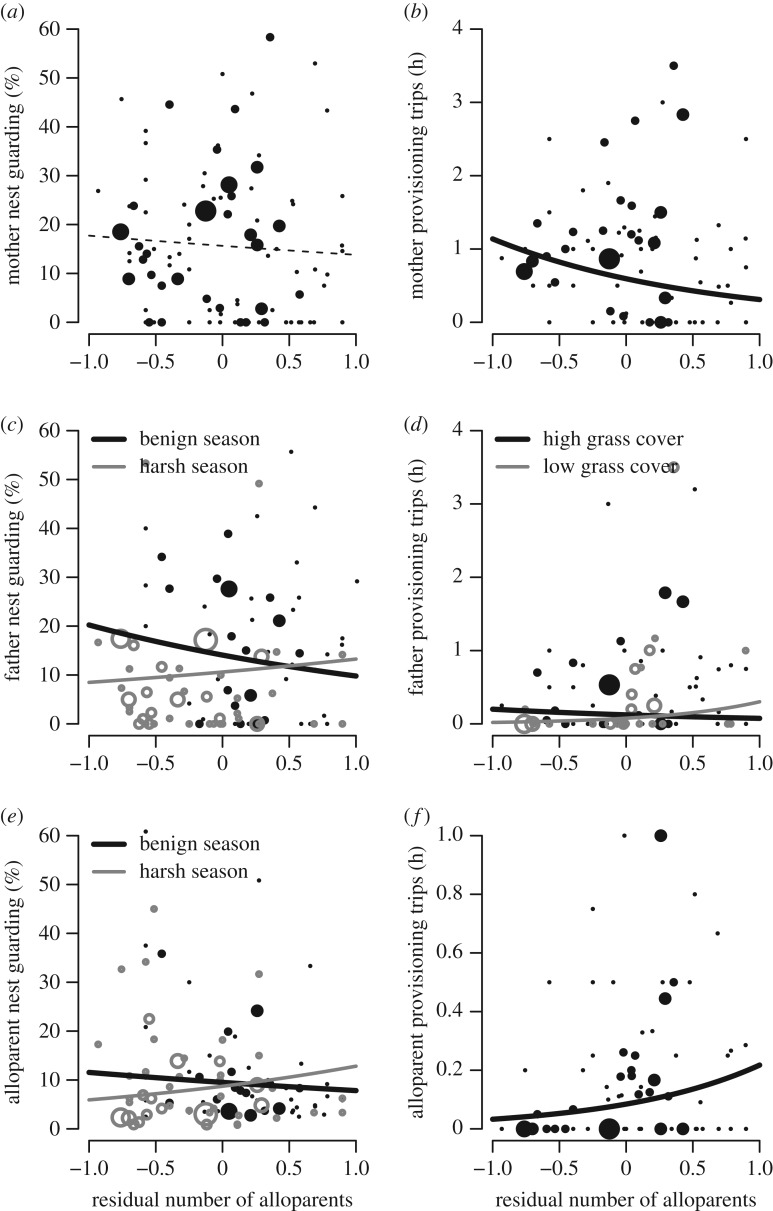
Mothers (*a*) did not alter their nest guarding as the number of alloparents increased, but (*b*) they did experience load-lightening in their provisioning rates. (*c*) Fathers experienced load-lightening in their nest guarding only in seasons of benign (black) relative to harsh breeding rainfall (grey). Conversely, (*d*) fathers increased their provisioning rates on low-quality territories (grey) compared to high-quality ones (black). Alloparents (*e*) increased nest guarding most in seasons of harsh (grey) relative to benign breeding rainfall (black), but (*f*) they increased provisioning rates similarly across environmental conditions. Lines represent GLMM model predictions (solid, statistically significant, dotted, non-significant); data points represent mean values for each mother in a given year, where symbol size is proportional to the sample size.

We also found that the cumulative guarding and offspring provisioning performed at a nest by all breeders and alloparents increased with the size of the alloparent contingent at that nest (see electronic supplementary material, S3 and table S3.2), providing a potential behavioural mechanism through which alloparents increase the number of nestlings fledged (figure 2). Cumulative nest guarding increased with the relative number of alloparents (figure 3) significantly more during harsh years with low breeding rainfall, though this increase remained positive during benign years with high breeding rainfall. Conversely, the cumulative provisioning rate at a nest increased with the number of alloparents but only when territory grass cover was low. On high-quality territories, provisioning rate did not change with the number of alloparents present. Similarly, the cumulative provisioning rate at a nest increased with the number of alloparents during benign breeding seasons relative to harsh ones. Together, these results indicate that the size of the alloparent contingent at a nest increases cumulative offspring care and significantly more so under harsh conditions, either with low breeding rainfall or low grass cover.

### 3.2. Load-lightening benefits

To determine whether individuals of different breeding roles experience load-lightening benefits and whether these benefits differ across temporal or spatial environmental conditions, we examined the behaviour of mothers, fathers and alloparents separately. First, we found that mothers did not alter their parental care behaviours with pre-breeding rainfall, breeding rainfall or grass cover (table 2). We also found that mothers did not experience load-lightening in their nest guarding behaviour (figure 3), suggesting that maternal nest guarding decisions are not affected by the presence of alloparents. However, mothers did experience load-lightening in their provisioning rates (table 2), as they performed less offspring provisioning as the number of alloparents increased (figure 3). Importantly, this provisioning load-lightening occurred under both harsh and benign rainfall conditions and low and high territory quality, as we found no significant interactions between environmental variables and the number of alloparents at a nest. Therefore, mothers experienced load-lightening benefits of having alloparents at the nest by reducing their provisioning rates similarly under both harsh and benign environments.

**Table 2.** Parameter estimates for three GLMMs examining how environmental conditions, the number of alloparents at a nest or the interaction between these variables shape time spent nest guarding in (A) mothers (*N* = 162), (B) fathers (*N* = 162) and (C) alloparents (*N* = 162). Asterisks highlight significant variables (*p* < 0.05). *p*-values and 95% Wald confidence intervals are presented in electronic supplementary material, table S3.4.

	(A) mothers		(B) fathers		(C) alloparents	
fixed effects	estimate ± s.e.	*Z*	estimate ± s.e.	*Z*	estimate ± s.e.	*Z*
intercept	−1.85 ± 0.15	−12.3*	−2.08 ± 0.19	−10.8*	−2.39 ± 0.12	−15.2*
pre-breed. rain	−0.07 ± 0.15	−0.48	−0.009 ± 0.16	−0.05	−0.02 ± 0.13	−0.13
breeding rain	0.18 ± 0.15	1.20	0.18 ± 0.11	1.71	0.06 ± 0.10	0.57
grass cover	−0.01 ± 0.11	−0.12	0.08 ± 0.13	0.64	0.05 ± 0.12	0.42
no. alloparents	−0.13 ± 0.24	0.51	−0.12 ± 0.18	−0.68	0.05 ± 0.20	0.24
pre-breed. rain × no. alloparents	0.13 ± 0.24	0.54	−0.11 ± 0.19	−0.56	−0.05 ± 0.16	−0.32
breeding rain × no. alloparents	−0.15 ± 0.22	−0.69	−0.37 ± 0.15	−2.43*	−0.37 ± 0.15	−2.48*
grass cover × no. alloparents	−0.05 ± 0.19	−0.27	0.07 ± 0.15	0.49	−0.09 ± 0.18	−0.51

random effects	variance ± s.d.	*N*	variance ± s.d.	*N*	variance ± s.d.	*N*
mother id	0.0000001 ± 0.0003	65			0.40 ± 0.63	65
father id			0.38 ± 0.62	59	0.13 ± 0.36	59
year	0.067 ± 0.26	13	0.19 ± 0.43	13	0.14 ± 0.37	13

Fathers did not alter their offspring care behaviour according to pre-breeding rainfall, breeding rainfall or territory grass cover (tables 2 and 3). Fathers did experience load-lightening in their nest guarding behaviour, but only in benign years with high breeding rainfall (figure 3). In harsh years with low breeding rainfall, fathers surprisingly increased their nest guarding as the number of alloparents increased at their nest. Therefore, fathers experienced load-lightening under benign but not harsh environmental conditions. Fathers also did not experience load-lightening in their nestling provisioning rates. Instead, paternal provisioning rates increased as the number of alloparents present increased (figure 3), particularly on low-quality territories. Fathers on high-quality territories still increased their offspring provisioning rates as the number of alloparents increased, but not as quickly as those on low-quality territories.

**Table 3.** Parameter estimates for three GLMMs examining how environmental conditions, the number of alloparents at a nest or the interaction between these variables shape nestling provisioning rates in (A) mothers (*N* = 130), (B) fathers (*N* = 130) and (C) alloparents (*N* = 130). Asterisks highlight significant variables (*p* < 0.05). *p*-values and 95% Wald confidence intervals are presented in electronic supplementary material, table S3.5.

	(A) mothers		(B) fathers		(C) alloparents	
fixed effects	estimate ± s.e .	*z*	estimate ± s.e.	*z*	estimate ± s.e.	*Z*
intercept	−0.52 ± 0.20	−2.66*	−2.25 ± 0.53	−4.22*	−2.47 ± 0.21	−12.0*
pre-breed. rain	−0.02 ± 0.11	−0.16	−0.62 ± 0.33	−1.87	0.35 ± 0.17	2.05*
breeding rain	−0.04 ± 0.09	−0.43	0.19 ± 0.23	0.84	0.23 ± 0.16	1.39
grass cover	−0.03 ± 0.12	−0.22	0.23 ± 0.26	0.90	−0.13 ± 0.19	−0.67
no. alloparents	−0.66 ± 0.16	−4.08*	0.14 ± 0.28	0.49	0.95 ± 0.30	3.16*
pre-breed. rain × no. alloparents	−0.05 ± 0.14	−0.35	−0.07 ± 0.25	−0.28	−0.62 ± 0.32	−1.92
breeding rain × no. alloparents	0.05 ± 0.12	0.46	0.41 ± 0.28	1.47	−0.15 ± 0.31	−0.49
grass cover × no. alloparents	−0.22 ± 0.14	−1.58	−0.94 ± 0.24	−3.96*	−0.27 ± 0.33	−0.84

random effects	variance ± s.d.	*N*	variance ± s.d.	*N*	variance ± s.d.	*N*
mother ID	0.50 ± 0.71	54			0.0000001 ± 0.0003	54
father ID			0.74 ± 0.86	51	0.62 ± 0.79	51
year	0.24 ± 0.49	12	1.79 ± 1.34	12	0.000001 ± 0.003	12

The average alloparent increased its nestling provisioning rate as pre-breeding rainfall increased (table 3). However, pre-breeding rainfall and grass cover were unrelated to alloparent offspring care behaviours (tables 2 and 3). Rather than showing load-lightening, however, alloparents increased the nest guarding and offspring provisioning they performed when there were relatively more alloparents at a nest. The positive relationship between an alloparent's nest guarding behaviour and the number of alloparents increased more rapidly in harsh years with low breeding rainfall (figure 3). This was no relationship between alloparent nest guarding and the size of the alloparent contingent during benign years with high breeding rainfall. Conversely, the positive relationship between an alloparent's provisioning rate and the number of alloparents at a nest was consistent across all spatial and temporal environmental conditions (interactions between the number of alloparents and environmental variables were not significant; table 2).

## 4. Discussion

This study tested competing hypotheses for why cooperatively breeding species are found more frequently in unpredictable environments—the hard life hypothesis, which argues that alloparents provide reproductive or load-lightening benefits only under harsh conditions, and the temporal variability hypothesis, which argues that alloparents provide reproductive or load-lightening benefits under both harsh and benign conditions. In support of the temporal variability hypothesis, we found that superb starling alloparents provided current reproductive benefits equally in harsh and benign conditions, both in terms of rainfall (temporal environmental variation), as well as for territory quality (spatial environmental variation). This is similar to results found in banded mongooses (*Mungos mungo*) and laughing kookaburras (*Dacelo novaeguinieae*)—two species found in similarly unpredictable semi-arid environments—as alloparents increased reproductive success independently of rainfall and territory quality, respectively [20,21]. Conversely, several studies have found that alloparents increase reproductive success only during unfavourable (i.e. hard life hypothesis) [7,16] or favourable environmental conditions [19]. Although future work examining how and why reproductive benefits of alloparental care change across environmental conditions will be necessary to reconcile current discrepancies in the literature, one possible explanation is that the frequency of harsh versus benign years differs among species and locations. For example, droughts and other periods of low rainfall may be more frequent than periods of high rainfall in the African savannah or Australian outback compared to the Mediterranean climates of North America or Europe [40].

To our knowledge, this study is the first to simultaneously explore whether the reproductive and load-lightening benefits of cooperative breeding trade off with one another across spatio-temporal environmental conditions. We found that in addition to reproductive benefits, superb starling mothers experienced load-lightening at their nests under both harsh and benign environmental conditions. Previous work in cooperatively breeding chestnut-crowned babbler (*Pomatostomus ruficeps*) [41] and campo flickers (*Colaptes campestris campestris*) [42] found that breeders can simultaneously gain reproductive and load-lightening fitness benefits of having alloparental care. For example, babbler mothers only partially decreased their provisioning rate with every additional alloparent present, which allowed for the cumulative provisioning at the nest to increase with the number of alloparents while simultaneously providing load-lightening for the mother. Therefore, superb starling mothers face a twofold advantage to recruiting alloparents, as alloparents increase a breeder's current reproductive success while also reducing potential costs of parental care in favour of the mother's own condition or survival. This result suggests that reproductive and load-lightening benefits do not trade off with one another in superb starlings. The reproductive and load-lightening benefits of cooperation have also been simultaneously examined in the acorn woodpecker (*Melanerpes formicivorus*), where breeders do not gain load-lightening benefits and instead maintain similar provisioning rates regardless of group size [8]. Breeders and alloparents in this species both increased their provisioning rates under benign environmental conditions [8], and, therefore, reproductive success also increased in benign years [19]. These studies [8,19,41] along with our own highlight interspecific differences in whether breeders favour reproductive or load-lightening benefits when aided by alloparents. Theoretical models suggest that group size, relatedness and the cost of care will affect whether reproductive versus load-lightening benefits are favoured [22,43], but future theoretical and empirical work should consider the impact of environmental variability on this trade-off.

Our results did not support the hard life hypothesis because we found no evidence that the reproductive or load-lightening benefits of having more alloparents were elevated exclusively under harsh conditions. Instead, we found that mothers experienced load-lightening benefits under both harsh and benign spatio-temporal conditions, and fathers experienced load-lightening in their nest guarding behaviour only under benign rainfall conditions, supporting the temporal variability hypothesis. We also found that under harsh conditions, fathers and alloparents increased their nest guarding and provisioning rates with the relative number of alloparents present. To our knowledge, no other study has found evidence for load-lightening exclusively under benign conditions. Since superb starling fathers perform less offspring care relative to mothers [24], reproduction may not be as costly for fathers relative to breeding females. Therefore, it is possible that fathers gain fewer fitness benefits by trading off current versus future fitness benefits than do mothers [22], although mothers simultaneously gain both types of benefits. A surprising finding of this study—that fathers and alloparents may *increase* offspring care with the number of alloparents—has been documented in only one other study. Breeders of the azure-winged magpie (*Cyanopica cyanus*) increased their investment in offspring care behaviour when joined by alloparents [17]. Much like superb starlings, nest failure in the azure-winged magpie is primarily driven by predation rather than offspring starvation [17]. Valencia and colleagues [17], therefore, suggested that a larger contingent of alloparents may increase the value of the current brood for the breeders, particularly in species where alloparents provide protection against predators [43]. In turn, fathers and alloparents may be more motivated to invest in offspring care when a brood is most likely to succeed (i.e. when more alloparents are present). Why fathers and alloparents would respond to changes in brood value associated with the size of the alloparent contingent at a nest while mothers invest similarly across broods requires further study, particularly because mothers can manipulate brood value based upon the number of alloparents present and environmental conditions in other cooperatively breeding species [44,45].

Finally, an alternative to this model of differential brood value is that fathers and alloparents socially reinforce each other's contributions to offspring care, either passively or actively. Passively, individuals may form flocks or follow conspecifics during feeding trips or guarding bouts, which could lead to an increase in the frequency of provisioning trips or time spent guarding in larger groups. Superb starlings often form foraging flocks during the breeding and non-breeding season [46], and they typically travel to and from the nest in small groups (S. Guindre-Parker and D.R. Rubenstein 2013--2016, personal observations), which lends support to this idea. Since harsh conditions also favour flocking in some bird species [47], superb starlings may remain closer together in harsh years and/or on low-quality territories, which would be consistent with this result. Alternatively, group members may enforce alloparent contributions to offspring care via aggressive behaviours or punishment [48], such that larger groups enforce offspring care to a greater degree. In superb starlings, alloparents in larger social groups have elevated testosterone which supports the idea that social conflict or competition may be elevated in these larger groups [49]. It remains unclear whether alloparents are punished for performing too little offspring care in superb starlings, and whether offspring care behaviour is more strongly reinforced under harsher conditions.

We found that individuals altered their offspring care behaviour according to breeding role, environmental conditions and the number of alloparents present. However, we were unable to determine whether behavioural load-lightening translated into future fitness benefits. This is because superb starlings frequently skip seasons where they neither breed nor help care for young [24], and as a result, re-sighting probabilities and breeding status in the following season are unreliable indicators of fitness. Determining whether load-lightening impacts future fitness requires lifetime monitoring. Since our dataset is only now beginning to encompass the lifespan of individual superb starlings (i.e. who live at least 14 years [24]), examining the fitness consequences of load-lightening will require additional years of data collection. Nevertheless, our results indirectly support the hypothesis that load-lightening may have future fitness benefits for two reasons. First, load-lightening should only arise when the benefit of this behaviour outweighs the benefit of maintaining additive care [15]. In superb starlings, cumulative offspring care at a nest increases reproductive success (S. Guindre-Parker and D.R. Rubenstein 2017, unpublished data), so we would not expect breeders to reduce their care unless they gained future fitness benefits that compensated for this loss in current reproductive success. Second, the magnitude of load-lightening observed in our study is similar to the magnitude observed in other species where future fitness benefits were documented (i.e. a twofold difference in provisioning or nest guarding with the smallest relative to largest contingent of alloparents) [13,50]. Together, this evidence suggests that load-lightening measured in our study system is likely to be biologically meaningful. Nevertheless, it remains possible that load-lightening occurs in the absence of future fitness benefits, as is the case for apostlebirds (*Struthidea cinerea*) [9,51].

In conclusion, our results are most consistent with the temporal variability hypothesis, though this hypothesis is formulated broadly. That is, the temporal variability hypothesis makes no distinction as to whether the benefits of alloparental care are (i) greater under harsh conditions but still present under benign conditions, (ii) greater under benign conditions but still present under harsh conditions, (iii) lower under intermediate conditions but present under harsh and benign conditions, or (iv) similar across all environmental conditions (from harsh to intermediate to benign). In an attempt to develop this hypothesis out further, our results support a number of these possibilities within a single study system, as reproductive success and maternal load-lightening benefits were similar under both harsh and benign conditions, whereas paternal load-lightening benefits occurred under benign but not harsh conditions. In cases where breeding cooperatively provides similar benefits across all environmental conditions, it becomes challenging to differentiate the temporal variability hypothesis from a general helper effect. To do so would require comparing benefits of alloparental care among intraspecific social groups that experience different degrees of variability in their environments. Importantly, our study also demonstrates that reproductive and load-lightening benefits of cooperative breeding differ across breeding roles and may vary across environmental conditions. Surprisingly, we found no evidence for a trade-off between reproductive and load-lightening benefits of alloparental care, because superb starling mothers experience incomplete load-lightening. Nevertheless, future work examining the fitness benefits of load-lightening will need to compare these two types of benefits in the same fitness currency. Studies examining the fitness benefits of breeding cooperatively that do not account for differences in breeding role or variation in environmental conditions may not capture the complexities of offspring care decision rules that shape alloparental behaviour in cooperative breeders. We suggest that breeding cooperatively in fluctuating environments may provide organisms with the behavioural flexibility necessary to adjust their investment in offspring care relative to the behaviour of others in their social group or to environmental conditions in order to invest optimally in current breeding versus self-maintenance, particularly in systems where individuals cannot simultaneously gain both types of benefits. This is consistent with the ‘bet-hedging hypothesis', which argues that cooperative breeding behaviour evolves to reduce environmentally driven fecundity variance [26]. It is also consistent with studies of maternal egg investment in superb fairy-wrens (*Malurus cyaneus*), where the degree of nutrients with which mothers provision eggs is dependent upon the size of the alloparent contingent at the nest [44]. Thus, the flexibility to trade off investment in current reproduction and future fitness via offspring care decision rules represents a potential mechanism underlying patterns of environmentally driven reproductive variance, as well as explaining why cooperatively breeding species occur more frequently in unpredictable environments. Ultimately, considering how offspring care decision rules vary with different breeding roles and across fluctuating environmental conditions will provide new insights into how and why cooperative breeding behaviour is linked to ecology.

## Supplementary Material

ESM 1 - GuindreParker&Rubenstein - The number of alloparents at a nest is independent of group size

## Supplementary Material

ESM 2 - GuindreParker&Rubenstein - Estimating the number of alloparents by correcting for observation length

## Supplementary Material

ESM 3 - GuindreParker&Rubenstein - Results of extra GLMMs presented within the article

## Data accessibility

We have made data available in Dryad: http://dx.doi.org/10.5061/dryad.6vc12 [30].
