# Supplementary material for: Multiple benefits of alloparental care in a fluctuating environment
Source: R Soc Open Sci. 2018 Feb 21;5(2):172406. doi: 10.1098/rsos.172406 (PMC5830800; doi:10.1098/rsos.172406)
Supplement: ESM 1 - GuindreParker&Rubenstein - The number of alloparents at a nest is independent of group size [file rsos172406supp1.pdf]

ELECTRONIC SUPPLEMENTARY MATERIALS TO:

Multiple benefits of alloparental care in a fluctuating environment

Sarah GUINDRE-PARKER & Dustin R. RUBENSTEIN

Royal Society Open Science

ESM 1: The number of alloparents at a nest is independent of group size

The number of alloparents providing offspring care to a brood of young can be correlated to, or constrained by, group size in many cooperatively breeding systems. However, superb starlings do not follow this pattern because non-breeding members of these plural breeding social groups can forgo alloparental care. In other words, non-breeding individuals can make the decision to provide alloparental care or to forgo providing any alloparental care (termed non-breeder/non-helpers). Therefore, the common correlation observed between group size and the number of alloparents partaking in offspring is not seen in superb starlings (Table S1). Future work will be necessary to address why and when individuals choose to serve as alloparents rather than become non-breeder/non-helpers.

Table S1: Parameter estimates and 95% Wald confidence intervals for a GLMM examining how total group size shapes the number of alloparents at a nest ($N = 162$). The model assumes a negative binomial error distribution and nest ID as a random effect to account for re-nesting attempts. Asterisks highlight significant variables.

**Fixed Effects**	**Estimate ± SE**	***Z***	***P***	**95% Wald CI**	
Intercept	1.03 ± 0.29	3.55	<0.001*	0.46	1.60
Group size	0.004 ± 0.01	0.33	0.73	-0.02	0.03
**Random Effect**	**Variance ± SD**	**N**			
Nest ID	0.50 ± 0.71	126			
