# Supplementary material for: Multiple benefits of alloparental care in a fluctuating environment
Source: R Soc Open Sci. 2018 Feb 21;5(2):172406. doi: 10.1098/rsos.172406 (PMC5830800; doi:10.1098/rsos.172406)

ELECTRONIC SUPPLEMENTARY MATERIALS TO:

Multiple benefits of alloparental care in a fluctuating environment

Sarah GUINDRE-PARKER & Dustin R. RUBENSTEIN

Royal Society Open Science

ESM 2: Estimating the number of alloparents by correcting for observation length

In our study, nest predation events prevented further observation periods from being performed. Nests that are observed for a longer period of time typically represent nests that survive to a later stage of nestling development (i.e. with older nestlings). Nestling age and observation length are therefore likely to confound the number of alloparents observed at a nest; nests with older chicks typically have more numerous alloparents present, and longer observation periods are more likely to capture alloparental care by infrequent alloparents. Nest age and observation length are correlated, so to correct for these potential confounding factors we examined whether the number of alloparents observed at a nest increased with the cumulative observation time or the age of nestlings during the last observation performed at a nest. We built two GLMMs to predict the number of alloparents at a nest using a negative binomial error distribution and a random effect of nest ID, and selected the best model using an information theoretic approach. Briefly, the number of alloparents increased with the length of cumulative focal observations performed at a nest (Figure S2.1) as well as the age of nestlings (Figure S2.2). However, the model accounting for the length of focal observations was a better fit for our dataset ($AIC = 936.1$) relative to the model accounting for age of nestlings ($AIC = 950.2$) or the null model ($AIC = 968$). Therefore, we chose to calculate the residuals of the number of alloparents on cumulative observation length (Table S2) to get an index of the residual number of alloparents at a nest. A greater positive value indicates that the nest had a larger contingent of alloparents then nests observed for a comparable length of time, and vice versa. We checked whether nestling age could still account for significant variation in this index of residual alloparents using a GLMM with Gaussian error distribution, but found no significant correlation (estimate ± SE = 0.01 ± 0.007, $t = 1.54$, $N = 127$, $P = 0.12$). Similarly, the residual number of alloparents on observation length was strongly correlated with the residual number of alloparents on nest age (estimate ± SE = 0.85 ± 0.05, $t = 17.6$, $N = 127$, $P < 0.001$). These results suggest that correcting the number of alloparents observed by taking the residuals on observation length also satisfactorily accounts for variation in nest age.

Table S2: Parameter estimates and 95% Wald confidence intervals for a GLMM examining how the number of alloparents counted at a nest increases with the length of cumulative observation time at that nest (*N* = 162). The model assumes a negative binomial error distribution and nest ID as a random effect to account for re-nesting attempts. Asterisks highlight significant variables.

**Fixed Effect**	**Estimate ± SE**	***Z***	***P***	**95% Wald CI**	
Intercept	0.62 ± 0.12	5.34	<0.001*	0.39	0.85
Group size	0.09 ± 0.01	6.65	<0.001*	0.06	0.11
**Random Effect**	**Variance ± SD**	**N**			
Nest ID	0.31 ± 0.56	127			

Figure S2.1: The number of alloparents counted at a nest increased with the length of cumulative observation time at that nest.

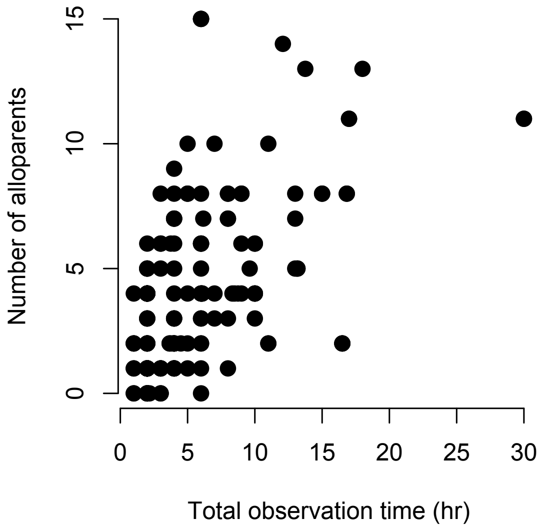

Figure S2.2: The number of alloparents observed at a nest increased with the age of nestlings during the last observation performed before either fledging or nestling death.

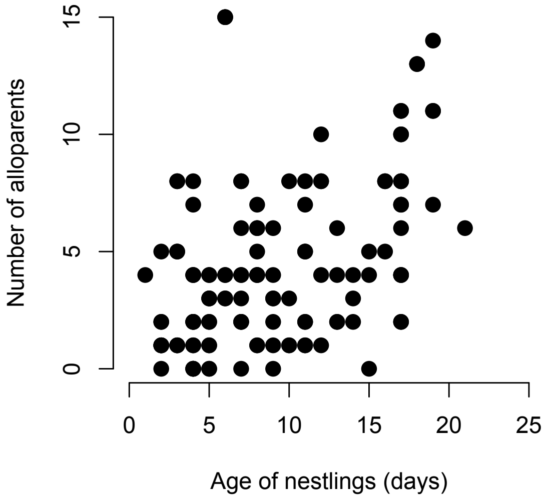

Supplement: ESM 2 - GuindreParker&Rubenstein - Estimating the number of alloparents by correcting for observation length [file rsos172406supp2.pdf]
