# Supplementary material for: Multiple benefits of alloparental care in a fluctuating environment
Source: R Soc Open Sci. 2018 Feb 21;5(2):172406. doi: 10.1098/rsos.172406 (PMC5830800; doi:10.1098/rsos.172406)
Supplement: ESM 3 - GuindreParker&Rubenstein - Results of extra GLMMs presented within the article [file rsos172406supp3.pdf]

ELECTRONIC SUPPLEMENTARY MATERIALS TO:

Multiple benefits of alloparental care in a fluctuating environment

Sarah GUINDRE-PARKER & Dustin R. RUBENSTEIN
Royal Society Open Science

ESM 3: Results of extra GLMMs presented within the article

Table S3.1: Parameter estimates and 95% Wald confidence intervals for three GLMMs examining how environmental conditions, the relative number of alloparents at a nest, or the interaction between these variables shape (**A**) clutch size (*N* = 162), (**B**) the number of nestlings that depredated (*N* = 134), or (**C**) the number of nestlings starved (*N* = 134). Asterisks highlight significant variables.

**A) Clutch size**					
**Fixed Effects**	**Estimate ± SE**	***Z***	***P***	**95% Wald CI**	
Intercept	1.16 ± 0.05	25.0	<0.001*	1.07	1.25
Pre-breeding rain	-0.03 ± 0.05	-0.58	0.56	-0.13	0.07
Breeding rain	0.03 ± 0.05	0.60	0.55	-0.07	0.13
Grass cover	0.009 ± 0.05	0.19	0.85	-0.08	0.10
No. alloparents	-0.07 ± 0.09	-0.84	0.40	-0.24	0.10
Pre-breeding rain * No. alloparents	-0.04 ± 0.08	-0.50	0.62	-0.19	0.11
Breeding rain * No. alloparents	-0.02 ± 0.08	-0.24	0.81	-0.18	0.14
Grass cover * No. alloparents	-0.02 ± 0.08	-0.23	0.82	-0.17	0.14
**Random Effects**	**Variance ± SD**		***N***		
Mother ID	0.0000001 ± 0.0003		65		
Father ID	0.0000003 ± 0.0006		59		
Year	0.0000002 ± 0.0004		13		

**B) Number of nestlings depredated**					
**Fixed Effects**	**Estimate ± SE**	***Z***	***P***	**95% Wald CI**	
Intercept	0.87 ± 0.10	8.88	<0.001*	0.68	1.07
Pre-breeding rain	-0.002 ± 0.11	-0.03	0.98	-0.22	0.21
Breeding rain	0.01 ± 0.11	0.12	0.90	-0.21	0.23
Grass cover	0.03 ± 0.08	0.38	0.71	-0.12	0.18
No. alloparents	-0.29 ± 0.16	-1.84	0.065	-0.59	0.02
Pre-breeding rain * No. alloparents	-0.02 ± 0.15	-0.15	0.88	-0.30	0.25
Breeding rain * No. alloparents	-0.10 ± 0.15	-0.68	0.50	-0.38	0.19
Grass cover * No. alloparents	-0.001 ± 0.12	-0.01	0.99	-0.23	0.23
**Random Effects**	**Variance ± SD**		***N***		
Mother ID	0.0000006 ± 0.0008		63		
Father ID	0.0000001 ± 0.0003		58		
Year	0.0000005 ± 0.0007		13		

**C) Number of nestlings starved**					
**Fixed Effects**	**Estimate ± SE**	***Z***	***P***	**95% Wald CI**	
Intercept	-1.75 ± 0.26	-6.74	<0.001*	-2.26	-1.24
Pre-breeding rain	0.08 ± 0.29	0.28	0.78	-0.48	0.67
Breeding rain	-0.25 ± 0.31	-0.79	0.43	-0.86	0.36
Grass cover	0.12 ± 0.28	0.42	0.67	-0.43	0.67
No. alloparents	0.22 ± 0.52	0.42	0.67	-0.80	1.24
Pre-breeding rain * No. alloparents	0.15 ± 0.48	0.31	0.76	-0.78	1.08
Breeding rain * No. alloparents	-0.77 ± 0.61	-1.28	0.20	-1.96	0.41
Grass cover * No. alloparents	-0.81 ± 0.61	-1.33	0.18	-2.00	0.38
**Random Effects**	**Variance ± SD**		***N***		
Mother ID	0.000001 ± 0.001		63		
Father ID	0.0000001 ± 0.0004		58		
Year	0.0000001 ± 0.0003		13		

Table S3.2: Parameter estimates and 95% Wald confidence intervals for two GLMMs examining how environmental conditions, the number of alloparents at a nest, or the interaction between these variables shape (A) time spent nest guarding ($N = 162$) and (B) the number of provisioning trips ($N = 130$) performed by all individuals cumulatively at a nest. Asterisks highlight significant variables.

**A) Cumulative guarding behaviour**					
**Fixed Effects**	**Estimate ± SE**	***Z***	***P***	**95% Wald CI**	
Intercept	-0.61 ± 0.13	-4.72	<0.001*	-0.87	-0.36
Pre-breeding rain	0.08 ± 0.09	-0.82	0.41	-0.26	0.10
Breeding rain	0.08 ± 0.06	1.23	0.22	-0.05	0.21
Grass cover	0.02 ± 0.06	0.36	0.72	-0.10	0.14
No. alloparents	0.47 ± 0.10	4.66	<0.001*	0.27	0.67
Pre-breeding rain * No. alloparents	0.12 ± 0.08	1.46	0.15	-0.04	0.29
Breeding rain * No. alloparents	-0.17 ± 0.08	-2.07	0.04*	-0.34	-0.009
Grass cover * No. alloparents	-0.00007 ± 0.08	0.00	1.00	-0.15	0.15
**Random Effects**	**Variance ± SD**		***N***		
Mother ID	0.01 ± 0.10		65		
Father ID	0.09 ± 0.29		59		
Year	0.17 ± 0.41		13		

**B) Cumulative provisioning behaviour**					
**Fixed Effects**	**Estimate ± SE**	***Z***	***P***	**95% Wald CI**	
Intercept	0.40 ± 0.15	2.73	0.009*	0.11	0.69
Pre-breeding rain	-0.11 ± 0.10	-1.06	0.29	-0.31	0.09
Breeding rain	0.10 ± 0.09	1.16	0.24	-0.07	0.26
Grass cover	0.03 ± 0.08	0.38	0.70	-0.13	0.20
No. alloparents	0.16 ± 0.13	1.19	0.23	-0.10	0.42
Pre-breeding rain * No. alloparents	-0.10 ± 0.12	-0.81	0.42	-0.34	0.14
Breeding rain * No. alloparents	0.26 ± 0.12	2.22	0.03*	0.03	0.49
Grass cover * No. alloparents	-0.32 ± 0.12	-2.65	0.008*	-0.56	-0.08
**Random Effects**	**Variance ± SD**		***N***		
Mother ID	0.12 ± 0.35		54		
Father ID	0.08 ± 0.29		51		
Year	0.15 ± 0.39		12		

Table S3.3: Parameter estimates, including *P*-values and 95% Wald confidence intervals for a GLMM examining how environmental conditions, the relative number of alloparents at a nest, or the interaction between these variables shape the number of nestlings that fledged ($N = 162$). Asterisks highlight significant variables ($P < 0.05$).

**Fixed Effects**	**Estimate ± SE**	***Z***	***P***	**95% Wald CI**	
Intercept	0.41 ± 0.12	3.30	<0.001*	0.17	0.65
Pre-breeding rain	-0.13 ± 0.12	-1.07	0.28	-0.36	0.11
Breeding rain	0.13 ± 0.10	1.21	0.23	-0.08	0.33
Grass cover	0.03 ± 0.11	0.30	0.76	-0.19	0.26
No. alloparents	0.82 ± 0.22	3.71	<0.001*	0.39	1.25
Pre-breeding rain * No. alloparents	0.11 ± 0.21	0.53	0.60	-0.30	0.52
Breeding rain * No. alloparents	0.32 ± 0.20	1.60	0.11	-0.07	0.71
Grass cover * No. alloparents	-0.20 ± 0.23	-0.90	0.37	-0.65	0.24
**Random Effects**	**Variance ± SD**		***N***		
Mother ID	0.0000007 ± 0.0008		65		
Father ID	0.0000001 ± 0.0003		59		
Year	0.000009 ± 0.003		13		

Table S3.4: Parameter estimates, including *P*-values and 95% Wald confidence intervals for three GLMMs examining how environmental conditions, the number of alloparents at a nest, or the interaction between these variables shape time spent nest guarding in (**A**) mothers (*N* = 162), (**B**) fathers (*N* = 162), and (**C**) alloparents (*N* = 162). Asterisks highlight significant variables (*P* < 0.05).

**A) Breeding Role: Mother**					
**Fixed Effects**	**Estimate ± SE**	***Z***	***P***	**95% Wald CI**	
Intercept	-1.85 ± 0.15	-12.3	<0.001*	-2.15	-1.56
Pre-breeding rain	-0.07 ± 0.15	-0.48	0.63	-0.36	0.22
Breeding rain	0.18 ± 0.15	1.20	0.23	-0.12	0.48
Grass cover	-0.01 ± 0.11	-0.12	0.91	-0.24	0.21
No. alloparents	-0.13 ± 0.24	-0.51	0.61	-0.60	0.35
Pre-breeding rain * No. alloparents	0.13 ± 0.24	0.54	0.59	-0.34	0.59
Breeding rain * No. alloparents	-0.15 ± 0.22	-0.69	0.49	-0.57	0.27
Grass cover * No. alloparents	-0.05 ± 0.19	-0.27	0.79	-0.43	0.33
**Random Effects**	**Variance ± SD**		***N***		
Mother ID	0.0000001 ± 0.0003		65		
Year	0.067 ± 0.26		13		
**B) Breeding Role: Father**					
**Fixed Effects**	**Estimate ± SE**	***Z***	***P***	**95% Wald CI**	
Intercept	-2.08 ± 0.19	-10.8	<0.001*	-2.46	-1.70
Pre-breeding rain	-0.009 ± 0.16	-0.05	0.96	-0.33	0.31
Breeding rain	0.18 ± 0.11	1.71	0.09	-0.03	0.39
Grass cover	0.08 ± 0.13	0.64	0.52	-0.17	0.33
No. alloparents	-0.12 ± 0.18	-0.68	0.50	-0.46	0.23
Pre-breeding rain * No. alloparents	-0.11 ± 0.19	-0.56	0.57	-0.48	0.26
Breeding rain * No. alloparents	-0.37 ± 0.15	-2.43	0.02*	-0.68	-0.07
Grass cover * No. alloparents	0.07 ± 0.15	0.49	0.63	-0.21	0.36
**Random Effects**	**Variance ± SD**		***N***		
Father ID	0.38 ± 0.62		59		
Year	0.19 ± 0.43		13		
**C) Breeding Role: Alloparent**					
**Fixed Effects**	**Estimate ± SE**	***Z***	***P***	**95% Wald CI**	
Intercept	-2.39 ± 0.12	-15.2	<0.001*	-2.70	-2.08
Pre-breeding rain	-0.02 ± 0.13	-0.13	0.90	-0.26	0.23
Breeding rain	0.06 ± 0.10	0.57	0.57	-0.13	0.25
Grass cover	0.05 ± 0.12	0.42	0.67	-0.18	0.28
No. alloparents	0.05 ± 0.20	024	0.81	-0.34	0.43
Pre-breeding rain * No. alloparents	-0.05 ± 0.16	-0.32	0.75	-0.37	0.26
Breeding rain * No. alloparents	-0.37 ± 0.15	-2.48	0.01*	-0.66	-0.08
Grass cover * No. alloparents	-0.09 ± 0.18	-0.51	0.61	-0.44	0.26
**Random Effects**	**Variance ± SD**		***N***		
Mother ID	0.40 ± 0.63		65		
Father ID	0.13 ± 0.36		59		
Year	0.14 ± 0.37		13		

Table S3.5: Parameter estimates, including *P*-values and 95% Wald confidence intervals for three GLMMs examining how environmental conditions, the number of alloparents at a nest, or the interaction between these variables shape nestling provisioning rates in (**A**) mothers ($N = 130$), (**B**) fathers ($N = 130$), and (**C**) alloparents ($N = 130$). Asterisks highlight significant variables ($P < 0.05$).

**A) Breeding Role: Mother**					
**Fixed Effects**	**Estimate ± SE**	***Z***	***P***	**95% Wald CI**	
Intercept	-0.52 ± 0.20	-2.66	0.008*	-0.90	-0.14
Pre-breeding rain	-0.02 ± 0.11	-0.16	0.87	-0.24	0.20
Breeding rain	-0.04 ± 0.09	-0.43	0.67	-0.21	0.14
Grass cover	-0.03 ± 0.12	-0.22	0.82	-0.26	0.21
No. alloparents	-0.66 ± 0.16	-4.08	<0.001*	-0.96	-0.34
Pre-breeding rain * No. alloparents	-0.05 ± 0.14	-0.35	0.73	-0.32	0.23
Breeding rain * No. alloparents	0.05 ± 0.12	0.46	0.65	-0.18	0.28
Grass cover * No. alloparents	-0.22 ± 0.14	-1.58	0.11	-0.49	0.05
**Random Effects**	**Variance ± SD**		***N***		
Mother ID	0.50 ± 0.71		54		
Year	0.24 ± 0.49		12		
**B) Breeding Role: Father**					
**Fixed Effects**	**Estimate ± SE**	***Z***	***P***	**95% Wald CI**	
Intercept	-2.25 ± 0.53	-4.22	<0.001*	-3.30	-1.21
Pre-breeding rain	-0.62 ± 0.33	-1.87	0.06	-1.27	0.03
Breeding rain	0.19 ± 0.23	0.84	0.40	-0.26	0.65
Grass cover	0.23 ± 0.26	0.90	0.37	-0.27	0.74
No. alloparents	0.14 ± 0.28	0.49	0.63	-0.41	0.68
Pre-breeding rain * No. alloparents	-0.07 ± 0.25	-0.28	0.78	-0.57	0.42
Breeding rain * No. alloparents	0.41 ± 0.28	1.47	0.14	-0.14	0.96
Grass cover * No. alloparents	-0.94 ± 0.24	-3.96	<0.001*	-1.40	-0.47
**Random Effects**	**Variance ± SD**		***N***		
Father ID	0.74 ± 0.86		51		
Year	1.79 ± 1.34		12		
**C) Breeding Role: Alloparent**					
**Fixed Effects**	**Estimate ± SE**	***Z***	***P***	**95% Wald CI**	
Intercept	-2.47 ± 0.21	-12.0	<0.001*	-2.87	-2.07
Pre-breeding rain	0.35 ± 0.17	2.05	0.04*	0.01	0.69
Breeding rain	0.23 ± 0.16	1.39	0.16	-0.09	0.55
Grass cover	-0.13 ± 0.19	-0.67	0.51	-0.49	0.24
No. alloparents	0.95 ± 0.30	3.16	0.002*	0.36	1.53
Pre-breeding rain * No. alloparents	-0.62 ± 0.32	-1.92	0.06	-1.25	0.01
Breeding rain * No. alloparents	-0.15 ± 0.31	-0.49	0.63	-0.77	0.46
Grass cover * No. alloparents	-0.27 ± 0.33	-0.84	0.40	-0.91	0.37
**Random Effects**	**Variance ± SD**		***N***		
Mother ID	0.0000001 ± 0.0003		54		
Father ID	0.62 ± 0.79		51		
Year	0.000001 ± 0.003		12		
